# An exploration of the links between parasites, trophic ecology, morphology, and immunogenetics in the Lake Tanganyika cichlid radiation

**DOI:** 10.1007/s10750-018-3798-2

**Published:** 2018-10-26

**Authors:** Britta S. Meyer, Pascal I. Hablützel, Anna K. Roose, Melinda J. Hofmann, Walter Salzburger, Joost A. M. Raeymaekers

**Affiliations:** 1 Zoological Institute, University of Basel, Vesalgasse 1, 4051 Basel, Switzerland; 2 Evolutionary Ecology of Marine Fishes, Helmholtz Centre for Ocean Research Kiel, GEOMAR, Düsternbrooker Weg 20, 24105 Kiel, Germany; 3 Present Address: Max Planck Institute for Evolutionary Biology, Max Planck Research Group Behavioural Genomics, August-Thienemann-Str. 2, 24306 Plön, Germany; 4 Laboratory of Biodiversity and Evolutionary Genomics, University of Leuven, Ch. Deberiotstraat 32, 3000 Louvain, Belgium; 5 Present Address: Flanders Marine Institute, Wandelaarkaai 7, 8400 Ostend, Belgium; 6 Department of Biodiversity and Evolutionary Biology, Museo Nacional de Ciencias Naturales, CSIC, Calle José Gutiérrez Abascal 2, 28006 Madrid, Spain; 7 Present Address: Museo de Zoología, Pontificia Universidad Católica del Ecuador, Av. 12 de Octubre 1076, Quito, Ecuador; 8 Present Address: Faculty of Biosciences and Aquaculture, Nord University, 8049 Bodø, Norway

**Keywords:** Parasites, Macroparasites, Trophic ecology, Morphology, Immunogenetics

## Abstract

Differences in habitat and diet between species are often associated with morphological differences. Habitat and trophic adaptation have therefore been proposed as important drivers of speciation and adaptive radiation. Importantly, habitat and diet shifts likely impose changes in exposure to different parasites and infection risk. As strong selective agents influencing survival and mate choice, parasites might play an important role in host diversification. We explore this possibility for the adaptive radiation of Lake Tanganyika (LT) cichlids. We first compare metazoan macroparasites infection levels between cichlid tribes. We then describe the cichlids’ genetic diversity at the major histocompatibility complex (MHC), which plays a key role in vertebrate immunity. Finally, we evaluate to what extent trophic ecology and morphology explain variation in infection levels and MHC, accounting for phylogenetic relationships. We show that different cichlid tribes in LT feature partially non-overlapping parasite communities and partially non-overlapping MHC diversity. While morphology explained 15% of the variation in mean parasite abundance, trophic ecology accounted for 16% and 22% of the MHC variation at the nucleotide and at the amino acid level, respectively. Parasitism and immunogenetic adaptation may thus add additional dimensions to the LT cichlid radiation.

**Electronic supplementary material:**

The online version of this article (10.1007/s10750-018-3798-2) contains supplementary material, which is available to authorized users.

## Introduction

Adaptive radiations of vertebrates belong to the most powerful model systems for the study of adaptation and diversification (Kornfield & Smith, 2000; Schluter, 2000; Berner & Salzburger, 2015). Sudden bursts of diversification often occur in newly emerging environments such as islands or lakes, which can offer colonizers a broad range of unoccupied niches in terms of habitat properties and food availability (Losos, 2010; Wagner et al., 2012). These bursts are promoted by natural selection on ecologically relevant traits such as body size and shape, trophic morphology, visual, acoustic and olfactory communication, and behavior (Rundell & Price, 2009; Schluter, 2009; Nosil, 2012). Sexual selection on mate choice behavior and ornamental traits is thought to further contribute to diversification (Salzburger, 2009; Wagner et al., 2012). It has been suggested that diversification in habitat use, trophic specialization, and the evolution of mating preferences represent the major axes of diversification underlying vertebrate adaptive radiations (Danley & Kocher, 2001; Streelman & Danley, 2003). These three axes may be tightly entangled, and pleiotropic interactions among genes or traits under both natural and sexual selection can accelerate speciation (“magic trait” principle) (Gavrilets, 2004; Servedio et al., 2011).

Parasites represent an important selection pressure, which is predominant in all living animals, and which may lead to strong evolutionary interactions (Decaestecker et al., 2013). Parasites have therefore also received attention in speciation research (Kaltz & Shykoff, 1998; Buckling & Rainey, 2002; Summers et al., 2003; Eizaguirre & Lenz, 2010; Karvonen & Seehausen, 2012). Parasite-induced speciation requires divergent parasite communities, adaptation to these parasite communities, and evolution of reproductive isolation (Karvonen & Seehausen, 2012). Specialization in habitat use has been shown to significantly affect exposure to parasites (Johnson et al., 2009; Matthews et al., 2010; Karvonen et al., 2013, 2018), and shifts in trophic niche may lead to changes in parasite transmission (Knudsen et al., 2010; Hablützel et al., 2017; Karvonen et al., 2018). Following adaptive divergence in allopatry, exposure of migrants to novel parasite communities may lead to reduced fitness and promote the evolution of prezygotic barriers (Nosil et al., 2005; MacColl & Chapman, 2010). Similarly, hybrids occurring in intermediate habitats or migrants between parental habitats face selective pressure of both parental parasite communities and hence could suffer from higher infection burdens (Hjältén, 1998; Fritz et al., 1999; Wolinska et al., 2006). Furthermore, introgression can create changes in the genetic background of resistance in hybrid offspring and thus increase parasite susceptibility. The reduced individual fitness can consequently lead to post-zygotic reproductive isolation (Karvonen & Seehausen, 2012). However, in some cases hybridization can lead to increased evolutionary adaptability due to additive effects of both parental species (Fritz et al., 1999; Seehausen, 2004). Finally, magic traits simultaneously involved in parasite defense and mate choice can further mediate adaptive divergence and the evolution of reproductive isolation (MacColl, 2009). Parasite burden can influence mating cues such as coloration and thus mating preferences (Hamilton & Zuk, 1982; Maan et al., 2008). This happens because male condition and disease resistance are under sexual selection, as infected individuals may appear less attractive as prospective mates (Adamo & Spiteri, 2009; Beltran-Bech & Richard, 2014).

Defense mechanisms against parasites range from behavioral mechanisms such as avoidance and cleaning behavior to molecular mechanisms. Among the latter, vertebrates have evolved a highly specific and efficient immune system based on antigen recognition referred to as adaptive immunity (Cooper & Alder, 2006; Flajnik & Kasahara, 2010). This system includes the major histocompatibility complex (MHC). Some proteins encoded by genes of the MHC play a central role by binding and presenting parasite-derived antigens to T cell receptors (Janeway, 2005). As antigen-binding can be highly specific, MHC genes evolved into one of the most polymorphic gene families of the vertebrate genome (Garrigan & Hedrick, 2003). In teleost fishes, the MHC is spread over three chromosomes. One chromosome contains the MHC class I, whereas the MHC class II loci are spread across two different chromosomes, called class II*a* and class II*b* genes. Each of these regions encompasses two separate subclasses of genes, the MHC class II A and B genes, coding for two different chains within the MHC molecules. In teleost fishes, the class II*a* genes are more conserved than the class II*b* genes. In cichlids, both groups of genes have oftentimes undergone several duplication events (Sato et al., 2012; Hablützel et al., 2013; Hofmann et al., 2017). MHC evolution is typically characterized by high standing variation (Sommer, 2005), positive and balancing selection (Hughes & Nei, 1988, 1989; Bernatchez & Landry, 2003), gene duplication and loss (Málaga-Trillo et al., 1998; Nei & Rooney, 2005), and extensive trans-species polymorphism (Klein et al., 2007). Interestingly, the MHC has been shown to act as a mate choice cue in several vertebrate species (Milinski, 2006; Piertney & Oliver, 2006; Kamiya et al., 2014). Due to this pleiotropic role of the MHC, diverging host populations adapting to contrasting parasite communities might simultaneously evolve reproductive isolation (Eizaguirre et al., 2009; Eizaguirre & Lenz, 2010).

The adaptive radiations of cichlids from the three East African Great Lakes (Lake Tanganyika, Victoria, and Malawi) represent important model systems to unravel causes of biological diversity and mechanisms of diversification (Kocher, 2004; Salzburger & Meyer, 2004; Seehausen, 2006; Salzburger, 2009, 2018; Maan & Seehausen, 2011). The approximately 250 cichlid species from Lake Tanganyika (LT) are genetically and morphologically very diverse and consist of 12–16 different morphological lineages (Salzburger et al., 2002; Koblmüller et al., 2008; Meyer et al., 2016). The other two hotspots, Lake Victoria and Lake Malawi, harbor hundreds of cichlid species belonging to the haplochromine tribe (Salzburger & Meyer, 2004; Joyce et al., 2011; Wagner et al., 2013). This enormous biodiversity can be partially explained by the palaeohydrological history and ecological opportunities (Salzburger et al., 2014), combined with sexual selection (Wagner et al., 2012). Cichlids have been suggested to have diverged along the three aforementioned major axes of diversification (adaptation to macrohabitats, diversification along feeding gradients, and mating preferences) (Danley & Kocher, 2001), though the chronology of these stages of diversification is under debate (Muschick et al., 2014).

A number of studies have investigated the importance of parasites and immunogenetics in fish and cichlid diversification (Vanhove et al., 2016; Malmstrøm et al. 2016). First, differentiation in parasite communities has been related to differences in morphology, trophic ecology, and evolutionary history of cichlid host species (Blais et al., 2007; Hablützel et al., 2016; Hayward et al., 2017). At the intra-specific level, such differentiation has also been described between allopatric populations, suggesting that parasites may represent a divergent selective force promoting divergence in allopatry (Raeymaekers et al., 2013; Grégoir et al., 2015; Hablützel et al., 2016). Second, extensive diversity and variation in MHC gene pools has been observed among cichlid species and populations (Klein et al., 1993; Ono et al., 1993; Málaga-Trillo et al., 1998; Blais et al., 2007; Sato et al., 2012; Hablützel et al., 2013, 2016; Hofmann et al., 2017). Dissimilarities in parasite community composition concur with differentiation of MHC class II genes in closely related cichlid species living in sympatry (Blais et al., 2007), as well as in allopatric populations within species (Hablützel et al., 2016). These results suggest a role for immunogenetic adaptation in cichlids. Individual MHC diversity has also been linked to variation in fat reserves, suggesting a relevant role of the MHC for host body condition (Hablützel et al., 2014). Third, a study by Maan et al. (2008) suggested that parasite-mediated sexual selection might contribute to the divergence of female mating preferences for male coloration, strengthening reproductive isolation. Together, these results indicate that the major axes of diversification (adaptation to macrohabitats, diversification along feeding gradients, and mating preferences) are potentially associated with exposure to different parasites or shifts in infection risk.

While these studies consider the possibility of parasite-driven diversification in cichlids between populations and closely related species, no study has investigated the potential contribution of parasitism to a cichlid adaptive radiation. Here, we explore this possibility for the adaptive radiation of LT cichlids. We base our analysis on a sample of 32 species across the tribes, which is about 12% of the LT cichlid species diversity. We first evaluated macroevolutionary relationships between trophic morphology, trophic ecology, and parasitism within LT cichlids. We expected different macroparasite communities among species, possibly between algae and invertebrate feeders (i.e., species that shifted in diet), or between generalists and sand and rock dwelling species (i.e., species that shifted in habitat) (Hablützel et al., 2017; Hayward et al., 2017). Second, we tested whether the species evolved immunogenetic differences by screening several loci of teleost MHC class II *B* genes. To achieve these goals, data by Muschick et al. (2012) on the trophic morphology and ecology of LT cichlids were combined with new parasitological and immunogenetic data.

## Materials and methods

### Field sampling and laboratory work

#### Parasitological survey

For the parasitological survey, 23 cichlid species were screened for metazoan ecto- and endoparasites (Supplementary Table 1). Sampling was conducted at Toby’s place on the Zambian shoreline of Lake Tanganyika. While most fish species were obtained in August 2012, *Simochromis diagramma* (Günther, 1894) and *Haplotaxodon microlepis* (Boulenger, 1906) were captured in August 2011 and July 2013, respectively. One species, *Astatotilapia burtoni* (Günther, 1894), was obtained in July 2013 at Kapata, which is about 20 km southward. About 7–18 individuals per species (usually ten) were caught by chasing fish into standing nets (Supplementary Table 1). After capture, the fish were kept in tanks of 0.8 m × 0.8 m × 1.2 m depth or 0.8 m × 0.8 m × 2 m depth. Before usage, tanks were cleaned, dried, and filled with lake water.

All fish were dissected in the field within 4 days post-capture. The day of dissection (0, 1, 2, or 3 days after capture) was recorded in order to keep track of changes in parasitological parameters while the fish were kept in the tanks. Individual fish were killed with an overdose of MS222. The parasitological survey consisted of three parts. First, the outer surface and the mouth cavity of the fish were inspected for ectoparasitic monogeneans and crustaceans (copepods, branchiurans, isopods), bivalves, and any kind of helminthic cysts. Second, the four gill-branches on the left were dissected and stored in 100% analytical ethanol (EtOH), and later in the lab screened for ectoparasitic monogeneans, crustaceans (copepods and branchiurans), bivalves, and any kind of helminthic cysts. Third, fish were screened for intestinal monogeneans, digeneans, acanthocephalans, nematodes, and any kind of helminthic cysts. To do so, stomach, intestines, gall and urinary bladder were dissected and inspected in a petridish with lake water. Finally, the sex of the fish was determined by visual inspection of the genital papilla and gonad development.

The parasitological survey was performed with a stereomicroscope and by multiple observers. Observers were recorded in order to keep track of observer bias. A single observer screened the outer surface and the mouth cavity of the fish. The number of observers varied between years for gills and intestines (gills: two observers in each year; intestines: three, four, and one observer(s) in 2011, 2012, and 2013, respectively). All parasites were counted and identified to genus or class level and preserved as follows: Monogeneans were isolated using dissection needles and were either mounted on slides in ammonium picrate glycerine for further morphological characterization, or stored in 100% EtOH. Acanthocephalans and nematodes were stored in 80% EtOH, while intestinal monogeneans, branchiurans, copepods, any kind of helminthic cysts, bivalves, and unknown groups were stored in 100% EtOH.

#### Analysis of MHC diversity

MHC loci are traditionally designated by a three-letter code and an Arabic number (e.g., DFB2). Whereas the first letter (D) designates the MHC class II gene, the second and third letters indicate the genomic region (letter code A–Z) and the subclass (A or B), respectively. A consecutive Arabic number defines the locus identity. Here, we focused on the MHC class II genes of the subclass b and only on the genes for the beta chain, which are located in the five genomic regions defined by (Sato et al., 2012) and which were named DBB-DFB. The genetic diversity of the MHC class II *B* genes within and among different LT cichlid tribes was analyzed for 26 species (Supplementary Table 1). The sampling was conducted in the years 2007 and 2013 at the shoreline of Lake Tanganyika between Mpulungu and Kalambo River, independently from the parasite screening, using a standard sampling procedure (Muschick et al., 2012). From each species, between 5 and 16 individuals were used for genotyping, which allowed us to characterize the most common variants present in each species (Supplementary Table 1). The same fish samples were used as in Meyer et al. (2016).

Due to the extreme polymorphism of MHC genes, especially in cichlids (Klein et al., 1993; Figueroa et al., 2000; Blais et al., 2007; Hofmann et al., 2017), it is notoriously difficult to obtain primers that can amplify all MHC loci within a single species, and presumably impossible to obtain primers that amplify all MHC loci across a wide range of species like the ones in this study. For this reason, we opted to use primers that amplify a subset of MHC loci in all species that we sampled, allowing our results to be comparable across species despite not amplifying the full diversity of MHC. For the amplification of these cichlid MHC loci, the forward primer TU383 (CTCTTCATCAGCCTCAGCACA; annealing upstream at the end of exon 1) and the reverse primer TU377 (TGATTTAGACAGARKGKYGCTGTA; annealing in exon 2 at base pair 248) (Ono et al., 1993) were used. This primer pair is known to amplify intron 1 and exon 2 of up to 17 homology groups in cichlids (Málaga-Trillo et al., 1998). In another study, up to eight expressed putative loci were found with this primer set (Blais et al., 2007). This primer pair has been successful in amplifying MHC in a wide range of cichlid species, and therefore we proceeded to the PCR without further optimization. The PCR amplification of the MHC was conducted in a final volume of 25 µL of the Multiplex PCR Kit (Qiagen, Hombrechtikon, Switzerland). Normalized DNA of the different species and sixteen MHC specific barcoded fusion primers (0.1 µM of each primer) were added. Fusion primers were synthesized at Microsynth (Balgach, Switzerland): the forward fusion primer is composed of the template-specific forward primer, the B-Adaptor, and the respective TCMID1–10 barcodes. Reverse primer is composed of the template-specific reverse primer and the A-Adaptor. In order to obtain sufficient amplicon product for further sequencing steps, we utilized a high number of PCR cycles, which is generally not recommended because of the inherent possibility of artifact generations (Acinas et al., 2005; Lenz & Becker, 2008). Standardized PCR conditions started with an initial heat activation phase (necessary for the HotStarTaq DNA polymerase) of 95°C, and continued with 35 amplification cycles consisting of 30 s of denaturation at 94°C, 90 s of annealing phase at 60°C, and an extension phase of 90 s at 72°C. The PCR was terminated with a final extension phase of 10 min at 72°C. The PCR products were purified with the magnetic bead system of Agencourt AMPure XP (Beckman Coulter, Nyon, Switzerland). The purification quality of the PCR products was assessed using the 2100 Bioanalyzer (Agilent, Basel, Switzerland) before the pyrosequencing step (454 with GS FLX system, Roche, conducted by Microsynth, Balgach, Switzerland). Our assessment does not employ all measures which can improve estimates of MHC diversity at the individual or species level (e.g., elimination of PCR artifacts through independent reaction assays, reconditioning PCR, increased elongation time, lower PCR cycles). However, the lack of such measures is not expected to affect our ability to compare patterns of MHC across a wide range of species, since any methodological bias is expected to be similarly distributed across the tribes.

### General sequence handling

First, the generated raw reads (11,569 reads) were processed with Roche’s demultiplexing and converting tools (sffinfo, sfffile) and sequences of primer annealing sites were removed. For quality filtering, we applied a filter for too short reads (≤ 150 bp). We only allowed 1% of ambiguous bases (N) and filtered out low-quality sequences (mean ≥ 15). These sequences were imported into Geneious (6.1.6 Biomatters Ltd., www.geneious.com) and de novo assembled (with custom sensitivity: minimum overlap identity of 95% and maximum ambiguity 4 using all reads from one species. This resulted in contigs of single individuals with highly identical reads (pairwise identity: median 99.50%) and contigs of several individuals sharing these reads (pairwise identity: median 99.40%). The coverage ranged from 2 to 131 for single individual contigs and 2–337 reads for contigs originating from multiple individuals. We also kept low coverage contigs as we use our data for measuring genetic diversity among tribes and not for investigating functionality or selection processes (indicated with suffix “low” in the alignment). However, if more than 3 bp of a read were different than the rest of the contig, the read was excluded. Also singletons, which differed dramatically (≥ 10 mutations) to other contigs, were removed from the data set (reads *N* = 517). Consensus sequences were generated within Geneious using 50% strict rule from each contig and for each individual. Most homopolymer regions were correctly called with these settings. Ambiguous positions were coded according to IUPAC rules. The obtained variants were aligned using MAFFT (–auto; 200PAM/k = 2, 1.53 open penalty/0.123 offset) (Katoh & Standley, 2013), and insertions of ambiguous positions, homopolymers, and misalignments were manually checked. This resulted in an alignment of 751 base pairs containing both intronic and exonic regions. A blast search of the alleles led to the exclusion of further sequences (removed contigs *N* = 266). In a next step, we shortened the alleles to exon 2 only, in order to (i) reduce our data set to coding nucleotides and (ii) to reduce the amount of missing data and ambiguities. This resulted in a total number of 844 MHC exon 2 variants of 160 bp.

### Specific data analysis

We here limit our statistical inference to the comparison between tribes (relative to the variation among species within tribes), rather than to a comparison among species (relative to the variation among individuals within species). For this exploratory study this is an appropriate choice, since the tribes represent the most important evolutionary branches of the LT radiation. As such, within our dataset representing a sample of 32 species across the tribes, we consider the species level (rather than the individual level) as the statistical unit. Analyses first aimed at the comparison of MHC diversity and parasite infection levels. Subsequently, in order to evaluate to what extent infection levels and immunogenetic divergence mirror adaptive radiation, we explore the relationships of these data with data on body shape, trophic morphology (pharyngeal jaw shape), and trophic ecology (diet and stable isotope signatures), available from Muschick et al. (2012) (Supplementary Table 1).

#### MHC diversity and differentiation

Immunogenetic diversity per species and immunogenetic differentiation between species was estimated with the software package MEGA (v.7) (Tamura et al., 2011). This was done for a set of 844 MHC variants, i.e., after excluding species with a very low amount of total MHC reads (Supplementary Table 1). Immunogenetic diversity was quantified as the average evolutionary divergence over sequence pairs within species. Ambiguous positions were eliminated in a complete comparison. The average evolutionary diversity was then subjected to a Kruskal–Wallis ANOVA to test for differences in MHC diversity between tribes.

In order to quantify immunogenetic differentiation, we calculated genetic distances between species on the basis of MHC variants of each species as a between group average. Specifically, we estimated the distance (uncorrected p-distance, complete deletion of missing data in the comparison) of the nucleotides of the exon (first, second, and third codon together; 143 positions). In addition, we calculated the distance of amino acid sequences using the Jones–Taylor–Thornton (JTT) model. This empirical substitution model corrects for multiple substitutions based on a model for amino acid substitutions using the substitution-rate matrix (Jones et al., 1992).

#### Phylogenetic distances

In order to account for the phylogeny in the analyses (see below), phylogenetic distances between the species were quantified by calculating genetic distances (uncorrected p-distances, pairwise deletion) based on sequences of 42 nuclear genes (17,545 nucleotides) from Meyer et al. (2015).

#### Parasites versus trophic ecology, morphology, and isotope signatures

The analyses of infection levels were performed in the statistical package R (R Core Team, 2014). Prevalence and mean abundance were calculated for each group of parasites and each host species following the terminology of Rózsa et al. (2000). A MANOVA was used to test for differences in infection levels (quantified either as prevalence or mean abundance) between cichlid tribes for all parasite groups together. Subsequently, Kruskal–Wallis ANOVAs were used to test for differences in infection levels among the tribes for each parasite group separately.

The level of covariation across cichlid species between infection levels on the one hand and body shape, trophic morphology, diet and stable isotope signatures on the other hand was investigated by a Spearman rank correlation analysis. Data on body shape, trophic morphology, diet, and stable isotope signatures, available from Muschick et al. (2012) (Supplementary Table 1), were included in the analyses as follows. Body shape was included as the two first principal components of body shape variation (Body1 and Body2), as calculated by Muschick et al. (2012) from a geomorphometric analysis. Likewise, trophic morphology was included using the two first principal components of lower pharyngeal jaw shape variation (LPJ1 and LPJ2), again as calculated by Muschick et al. (2012). Diet was included as proportional prey data, as well as the two first principal components calculated from these data (Prey1 and Prey2). The isotope signatures included carbon and nitrogen stable isotopes (δ13C and δ15N), which are a proxy for trophic ecology (Boecklen et al., 2011; Muschick et al., 2012). In particular, δ13C values in LT cichlids were found to be correlated with body shape clusters, whereas δ15N values correlate with the shape of the lower pharyngeal jaw. As such the δ13C and δ15N, respectively, reflect variation between macrohabitats (e.g., benthic versus pelagic) and the relative trophic level of an organism.

To further investigate how much of the variation in infection levels (combining all parasite groups) among cichlid species could be explained by body shape, trophic morphology, diet, or isotopes, we performed a redundancy analysis (RDA). RDA is a canonical extension to PCA in which the principal components produced are constrained to be linear combinations of a set of predictor variables (Legendre & Legendre, 2012). It enables the identification of the best ordination model that describes parasite community similarities among cichlid species. In order to account for phylogeny in this analysis, the set of predictor variables also included the first two dimensions of a classical multidimensional scaling (CMDS) analysis on the phylogenetic distances. RDA analysis was performed with the R library vegan. Significance of the proportion of variation in infection levels explained by each source of information was calculated and tested for significance using 1000 random permutations. For each source of information, the RDA analysis was preceded by a forward selection procedure as implemented in the “packfor” package in R (Dray et al., 2009, 2012). Forward selection corrects for highly inflated type I errors and overestimated amounts of explained variation.

#### MHC versus parasites, trophic ecology, morphology, and isotope signatures

All analyses in this section were performed separately for the MHC-based genetic distances based on the exon 2 nucleotide sequences (143 bp) and the translated amino acid sequences (using JTT model). First, a permutational ANOVA on the MHC-based genetic distances was performed to test for significant differences in MHC profiles between tribes. A CMDS analysis was then used to convert these MHC-based genetic distances into a set of coordinates (dimensions) for further analyses. The first (MHC dimension 1) versus second (MHC dimension 2) dimension of these CMDS analyses were first plotted to visualize the immunogenetic differences between cichlid species and tribes. Variation in MHC profiles among species was further visualized in a cluster diagram using the UPGMA criterion based on MHC distances. We then investigated the relationships across cichlid species between these MHC dimensions and infection levels, body shape, trophic morphology, diet, and stable isotope signatures by means of a Spearman rank correlation analysis. Finally, RDA analyses were performed to investigate how much of the variation in MHC dimensions (MHC dimension 1 and MHC dimension 2) could be explained by infection levels, body shape, trophic morphology, diet, isotopes, or phylogeny. As above, significance of the proportion of variation in MHC dimensions explained by each source of information was calculated and tested for significance using 1000 random permutations, and for each source of information, the RDA analysis was preceded by a forward selection model procedure. Note that the subset of cichlid species for which parasite data were available was smaller than the subset for which body shape, trophic morphology, diet, and stable isotope data were available. Therefore, the Spearman rank correlation analysis as well as the RDA analyses were performed separately for these subsets.

## Results

### Parasites versus trophic ecology, morphology, and isotope signatures

**Fig. 1:**
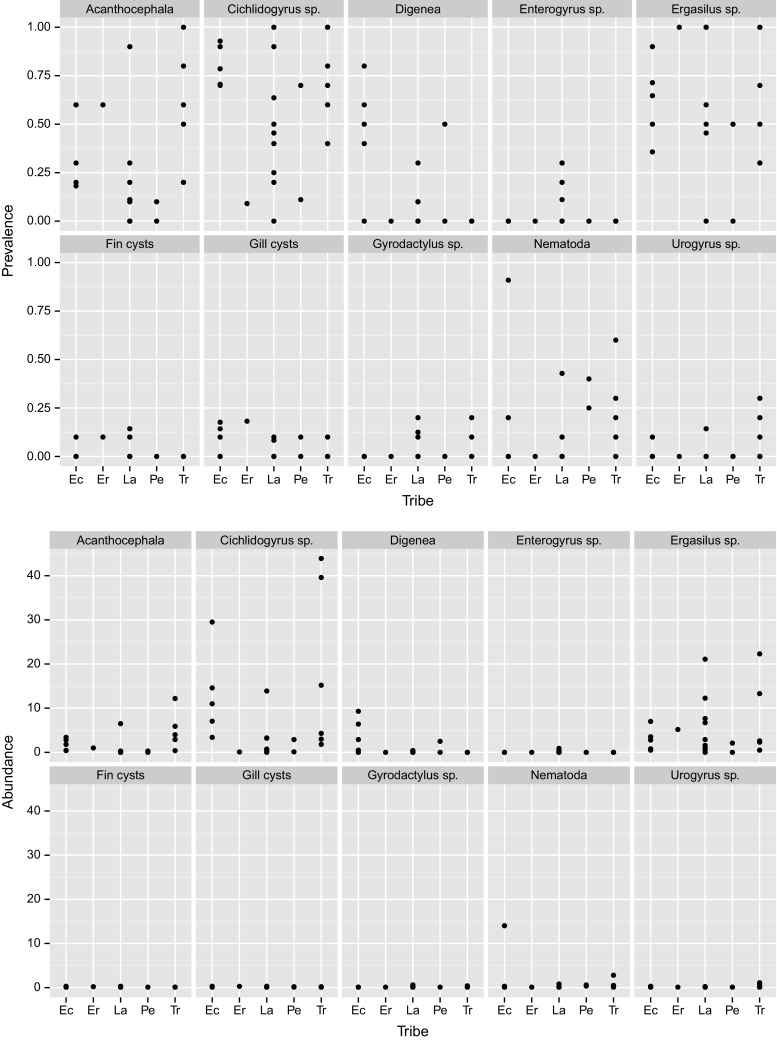
Prevalence (top) and mean abundance (bottom) of ten groups of endo- and ectoparasites by cichlid tribe. *Ec* Ectodini, *Er* Eretmodini, *La* Lamprologini, *Pe* Perissodini, *Tr* Tropheini

**Fig. 2:**
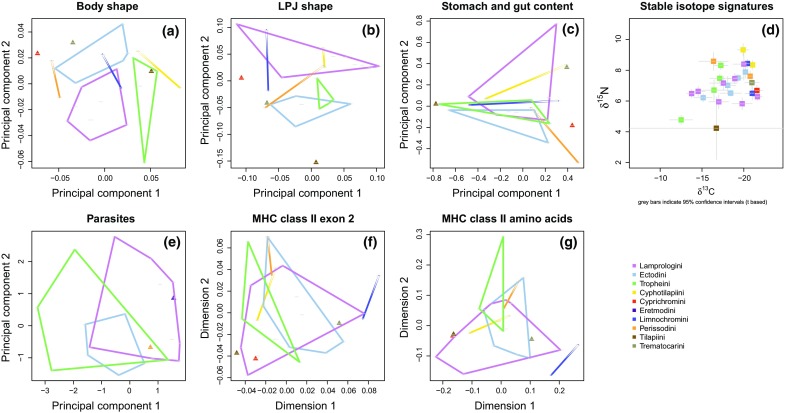
Two-dimensional representations based on principal component analyses (PCA) or classical multidimensional scaling (CMDS) of morphology, trophic ecology, isotope signatures, infection levels, and MHC-based genetic divergence in Lake Tanganyika cichlids. **a** PCA on body shape; **b** PCA on LPJ shape; **c** PCA on proportional stomach and gut contents; **d** stable isotope data (δ15N versus δ13C); **e** PCA on mean square-root transformed abundance of parasites; **f** CMDS on between-species MHC classII*b* B exon 2 genetic distances; **g** CMDS on between-species MHC classII*b* B amino acid distances. Filled triangles represent tribes for which only one species was analyzed; gray bars in **d** indicate t-based 95% confidence intervals. Plot **a–d** are based on data from Muschick et al. (2012)

MANOVA revealed significant differences between LT cichlid tribes for the prevalence of metazoan ecto- and endoparasites (Wilks’ lambda = 0.0066, *F*_40,28_ = 1.96, *P* = 0.0322). These differences were mainly due to the prevalence of acanthocephalans, which was high in Tropheini, intermediate in Ectodini, and low in Lamprologini and Perissodini, as well as to the prevalence of digeneans which was high in Ectodini (Table 1; Fig. 1; Supplementary Table 5). There was no multivariate difference between the tribes for the mean abundance of parasites (Wilks’ lambda = 0.027, *F*_40,28_ = 1.12, *P* = 0.38). However, univariate tests revealed high values for acanthocephalans and *Cichlidogyrus* sp. in Tropheini, intermediate values in Ectodini, and low values in Lamprologini and Perissodini, while the abundance of digeneans was high in the Ectodini (Table 1; Fig. 1; Supplementary Table 6). A two-dimensional (PCA-based) representation of parasite communities (Fig. 2) revealed partially non-overlapping parasite communities in the Lamprologini and Tropheini, while parasite communities in the Ectodini show similarities with both the Lamprologini and the Tropheini.

**Table 1:** Non-parametric (Kruskal–Wallis) ANOVA on the prevalence of metazoan ecto- and endoparasites between five Lake Tanganyika cichlid tribes

	Prevalence			Mean abundance		
	*χ* ^2^	*df*	*P* value	*χ* ^2^	*df*	*P* value
Endoparasites						
Acanthocephala	9.90	4	**0.0422**	12.53	4	**0.0138**
Nematoda	5.05	4	0.2821	4.88	4	0.2998
*Urogyrus*	4.46	4	0.3468	4.38	4	0.3572
*Enterogyrus*	7.13	4	0.1291	7.12	4	0.1295
Digenea	10.30	4	**0.0356**	10.43	4	**0.0338**
Ectoparasites						
Gill cysts	7.56	4	0.1092	5.98	4	0.2007
Fin cysts	4.99	4	0.2880	4.14	4	0.3874
*Gyrodactylus*	3.13	4	0.5358	3.10	4	0.5412
*Cichlidogyrus*	7.62	4	0.1063	12.29	4	**0.0153**
*Ergasilus*	4.00	4	0.4060	2.30	4	0.6811

Significant *P* values are in bold

**Fig. 3:**
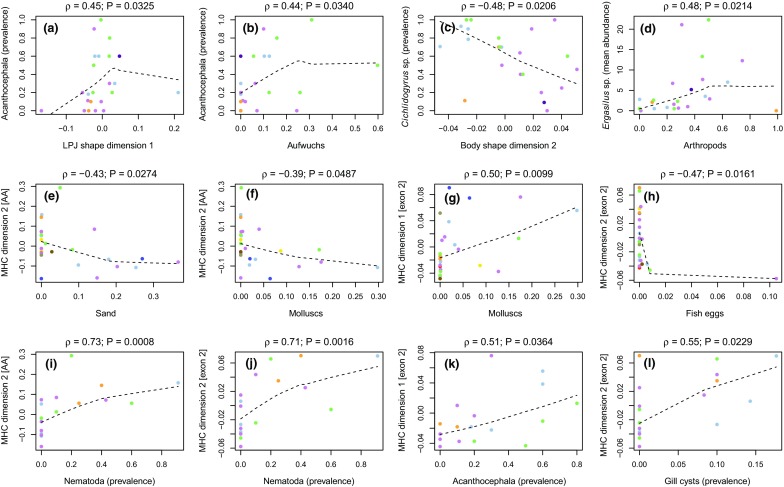
Relationships between infection levels and trophic ecology or morphology (**a**–**d**; 23 cichlid species), MHC divergence and trophic ecology (**e**–**h**; 26 cichlid species), and MHC divergence and infection levels (**i**–**l**; 17 cichlid species). Dashed lines were obtained with a lowess function. The colors distinguish species from different tribes according to the color scheme of Fig. 2

Spearman rank correlations revealed that infection levels across cichlid species were correlated with morphology and diet (Supplementary Table 2). For instance, the prevalence of acanthocephalans was correlated with LPJ shape (PC1; Fig. 3a) and increased with the proportion of aufwuchs in the diet (Fig. 3b), while the prevalence of *Cichlidogyrus* sp. correlated with body shape (PC2) (Fig. 3c). Spearman rank correlations with mean abundance confirmed these results (Supplementary Table 2), and also revealed an increase of *Ergasilus* sp. with the proportion of arthropods in the diet (Fig. 3d).

Forward selection followed by RDA identified a significant effect of body shape (PC2) on the entire parasite community, accounting for 15% of the variation in mean abundance after controlling for phylogenetic relationships (RDA: *F*_2,13_ = 2.72; *P* = 0.041). Other variables accounting for trophic ecology, morphology, or isotope signatures were not selected in these models. No models explained significant variation in prevalence.

### MHC versus parasites, trophic ecology, morphology, and isotope signatures

In 316 individuals from 26 different species we identified 844 variants in total, of which 388 are unique variants. Averaged across all tribes, we thus find 93.8 variants in total and 43.1 unique variants per tribe. Overall, we find an average of 14.9 variants per species, which was represented by on average 12.2 individuals. Per individual we have sequenced one to nine (mean 2.6) variants. The overall genetic distance within the three major tribes, namely the Lamprologini, the Ectodini, and Tropheini, was very similar (ranging around 0.2) (see Tables 2, 3). ANOVA did not reveal any significant differences in MHC diversity (based on average evolutionary divergence) among tribes (*χ*^2^ = 13.226, *df* = 8, *P* = 0.103).

**Table 2:** MHC variants within the species: species name and tribe; average evolutionary divergence (calculated over sequence pairs within species); number of total and unique variants; number of individuals sequenced; average, minimum, and maximum numbers of variants per species and individual; average MHC-based (genetic) distances of amino acids and nucleotides of each species to the other species

Species	Tribe	Divergence	Total	Unique	Ind.	Average	Min.	Max.	Distance, amino acids	Genetic distance, nucleotides
*Cyphotilapia frontosa* (Boulenger, 1906)	Cyphotilapiini	0.155	28	18	16	2.00	1	6	0.507	0.187
*Trematochromis benthicola* (Matthes, 1962)	Cyphotilapiini	0.159	15	6	7	2.14	1	5	0.516	0.196
*Cyprichromis leptosoma* (Boulenger, 1898)	Cyprichromini	0.178	56	27	11	5.09	1	9	0.556	0.202
*Aulonocranus dewindti* (Boulenger, 1899)	Ectodini	0.132	8	5	7	1.14	1	2	0.482	0.193
*Callochromis macrops* (Boulenger, 1898)	Ectodini	0.178	57	19	16	3.56	1	9	0.537	0.197
*Grammatotria lemairii* (Boulenger, 1899)	Ectodini	0.184	38	22	15	2.53	1	4	0.536	0.197
*Ophthalmotilapia ventralis* (Boulenger, 1898)	Ectodini	0.191	48	25	16	2.80	2	4	0.539	0.198
*Xenotilapia spiloptera* (Poll & Stewart, 1975)	Ectodini	0.176	50	17	16	3.13	1	5	0.548	0.196
*Astatotilapia burtoni* (Günther, 1894)	Tropheini	0.182	26	7	8	3.25	2	4	0.521	0.199
*Ctenochromis horei* (Günther, 1894)	Tropheini	0.207	34	18	15	2.27	1	4	0.571	0.212
*Lobochilotes labiatus* (Boulenger, 1898)	Tropheini	0.188	55	19	16	3.44	1	6	0.544	0.204
*Tropheus moori* (Boulenger, 1898)	Tropheini	0.196	27	16	14	1.93	1	4	0.528	0.203
*Lamprologus callipterus* (Boulenger, 1906)	Lamprologini	0.165	21	12	14	1.54	1	3	0.534	0.197
*Lepidiolamprologus elongatus* (Boulenger, 1898)	Lamprologini	0.186	73	31	16	4.56	1	8	0.542	0.199
*Neolamprologus modestus* (Boulenger, 1898)	Lamprologini	0.167	19	13	7	2.71	1	3	0.542	0.196
*Neolamprologus prochilus* (Bailey & Stewart, 1977)	Lamprologini	0.223	6	6	5	1.20	1	2	0.562	0.202
*Neolamprologus pulcher* (Trewavas & Poll, 1952)	Lamprologini	0.193	37	15	13	2.85	1	6	0.567	0.203
*Neolamprologus tetracanthus* (Boulenger, 1899)	Lamprologini	0.181	28	13	8	3.50	2	5	0.559	0.200
*Telmatochromis dhonti* (Boulenger, 1919)	Lamprologini	0.208	35	24	14	2.50	1	6	0.563	0.205
*Variabilichromis moorii* (Boulenger, 1898)	Lamprologini	0.174	24	10	14	1.71	1	3	0.510	0.193
*Gnathochromis permaxillaris* (David, 1936)	Limnochromini	0.162	46	6	16	2.88	1	5	0.560	0.199
*Limnochromis abeelei* (Poll, 1949)	Limnochromini	0.156	13	5	7	1.86	1	3	0.581	0.203
*Haplotaxodon microlepis* (Boulenger, 1906)	Perrissodini	0.187	20	8	12	1.67	1	3	0.525	0.204
*Perissodus microlepis* (Boulenger, 1898)	Perrissodini	0.16	32	9	14	2.36	1	4	0.514	0.192
*Oreochromis tanganicae* (Günther, 1894)	Oreochromini	0.205	13	10	7	1.86	1	4	0.551	0.207
*Trematocara nigrifrons* (Boulenger, 1906)	Trematocarini	0.153	35	27	12	2.92	1	5	0.501	0.189

**Table 3:** MHC variants across the major cichlid tribes of LT with number of included species, number, and average of variants (total and unique) and the genetic distance based on the exon sequences (uncorrected p-distance and absolute difference)

Tribe	Species included	Total # of variants	Average # of total variants	Unique # of variants	Average # of unique variants	Uncorrected p-distance of sequences within each tribe	Absolute # of base differences
Cyphotilapini	2	43	21.5	24	12	0.17	26.4
Cyprichromini	1	56	56	27	27	0.18	28.7
Ectodini	5	201	40.2	88	17.6	0.19	29.9
Tropheini	4	142	35.5	60	15	0.2	32.3
Lamprologini	8	243	30.4	124	15.5	0.19	30.9
Limnochromini	2	59	29.5	11	5.5	0.16	26.4
Perissodini	2	52	26	17	8.5	0.17	27.9
Oreochromini	1	13	13	10	10	0.2	32
Trematocarini	1	35	35	27	27	0.15	24.1

MHC dissimilarities between cichlid species could be attributed to tribe (nucleotide level of exon 2: *F*_8,16_ = 1.46; *P* = 0.013; *R*^2^ = 0.42; amino acid level: *F*_8,16_ = 2.52; *P* = 0.003; *R*^2^ = 0.56), but with a lower proportion of variation than for phylogenetic dissimilarities between species (*F*_8,16_ = 51.75; *P* = 0.001; *R*^2^ = 0.96). Accordingly, a two-dimensional (MDS-based) representation of MHC dissimilarities between cichlid species revealed partial overlap between the three largest tribes (Tropheini, Ectodini, and Lamprologini) at the nucleotide level of exon 2 as well as the amino acid level (Fig. 2f, g). The MHC profiles of the two species belonging to the Limnochromini were distinct from other tribes, especially at the amino acid level (Fig. 2g). The position of Limnochromini at the root of the MHC differentiation is confirmed by a (UPGMA-based) cluster analysis at the amino acid level, but not at the nucleotide level, where synonymous mutations are also taken into account (Supplementary Fig. 1).

Spearman rank correlations revealed relationships between the first and second MHC dimension and trophic ecology and infection levels, but not between MHC and morphology (Fig. 3; Supplementary Tables 3 and 4). At the amino acid level, MHC dimension 2 decreased with the proportion of sand (Fig. 3e) and molluscs (Fig. 3f) in the diet, and increased with the prevalence and mean abundance of nematodes (Fig. 3i). For the nucleotide level of exon 2, MHC dimension 1 increased with feeding on molluscs (Fig. 3g) and with the prevalence of acanthocephalans (Fig. 3k). MHC dimension 2 for nucleotide level of exon 2 decreased with feeding on fish eggs (Fig. 3h), and increased with infection levels of nematodes (Fig. 3j) and gill cysts (Fig. 3l).

Forward selection followed by RDA on MHC dimension 1 and MHC dimension 2 identified a significant effect of the prevalence of nematodes on MHC divergence after controlling for phylogenetic relationships. The model explained 30% of the variation at the nucleotide level (RDA: *F*_1,10_ = 5.15; *P* = 0.01). Yet, none of the infection parameters explained significant variation at the MHC amino acid level. Feeding on sand explained 16% of the MHC variation at the nucleotide level (RDA: *F*_1,21_ = 5.0; *P* = 0.006), while feeding on sand and fish eggs accounted for 22% of the MHC variation at the amino acid level (RDA: *F*_2,20_ = 4.05; *P* = 0.004). Variables quantifying morphology or isotope signatures did not explain significant variation at the MHC nucleotide or amino acid level.

## Discussion

In this study, we explore the relationships between parasite infection, trophic ecology, morphology, and immunogenetics in the LT cichlid radiation. Within such a large adaptive radiation of fishes, and with parasites as one of the most diverse taxa and the MHC as one of the most diverse genes, our assessment of the diversity at both levels is inevitably incomplete. The patterns that emerge from the comparison between these data layers are thus preliminary and must be interpreted with caution.

### Parasite diversity

Habitat adaptation and trophic adaptation have been proposed to be important drivers of the LT cichlid radiation, because the habitat and diet of the various cichlid species are strongly linked with morphology. For instance, Muschick et al. (2012) observed that habitat is associated with differences in body shape, while diet is associated with pharyngeal jaw morphology, a key trait for feeding on a specialized diet (from algae and biofilms to invertebrates and fish). In addition, carbon and nitrogen stable isotope signatures, which are classical indicators of the habitat and trophic position of an organism, also correlate with morphology (Muschick et al., 2012). In particular, δ13C values in LT cichlids are associated with body shape clusters characteristic for a benthic or pelagic lifestyle, and δ15N values correlate with the shape of the lower pharyngeal jaw. Stable isotope signatures thus reflect variation between macrohabitats as well as the relative trophic level of an organism.

In this study, we anticipated that habitat divergence and diet shifts might lead to exposure to different parasites and shifts in infection risk. If so, variation in morphology, diet, or stable isotopes among cichlid species should reflect differences in the composition of parasite communities. Accordingly, we observed that variation in body shape, LPJ shape, and individual prey items among species correlated either with overall parasite community composition or with the infection levels of individual parasite categories. For instance, LPJ shape correlated with the prevalence of acanthocephalans, and body shape accounted for 15% of the variation in the overall parasite community. Overall, different cichlid tribes featured partially non-overlapping parasite communities. These observations are not surprising given that variation in trophic-morphological traits enables cichlids to occupy different niches, which may harbor different parasites. Nevertheless, it has a major implication for the understanding of cichlid species diversification: adaptation to novel habitats or diets may as well require adaptation to different parasite environments. Because of the coexistence of many of the investigated hosts at the same locality, confounding effects arising as a consequence of geographical separation are minimal in this study.

This study is the first to investigate the assertion of parasite-driven species diversification across representative species from multiple tribes within an entire adaptive radiation. Recently, Baldo et al. (2017) came to a similar conclusion for gut bacteria, which significantly deviated between cichlid species with a carnivorous and herbivorous lifestyle. Overall, there are thus substantial indications that habitat and diet influence both the bacterial microbiota as well as parasitic macrobiota with the LT cichlid radiation. Previously, Hablützel et al. (2017) demonstrated that trophic divergence can also lead to divergence in parasite communities at younger branches of the LT radiation. Within the Tropheini, one of the tribes included in this study, species evolved from relatively unselective substrate browsing of aufwuchs to more specialized foraging strategies, such as selective combing of microscopic diatoms or picking of macro-invertebrates. This divergence entailed reduced ingestion of intermediate invertebrate hosts of acanthocephalans (i.e., “parasite escape”), hence potentially facilitating niche divergence (Hablützel et al., 2017). Possibly, the level of trophic specialization can also explain infection at the tribe level. For instance, the relatively indifferent feeders of aufwuchs including Tropheini and Ectodini had higher infection levels with acanthocephalans than the Lamprologini and Perissodini, who pick their food selectively. Yet, the explanation for differences in infection by other parasites such as digeneans or *Cichlidogyrus* sp. is more obscure, because the infectious stage of these parasite groups search for hosts actively. Overall, the knowledge of the biology of the large diversity of LT fish parasites remains poor (Vanhove et al., 2016), and thus, why certain cichlid tribes are more infected with specific parasite groups than others is unclear.

Interestingly, the best predictor of parasite load within the LT cichlid radiation among a set of host characteristics was the number of host species that a particular host may encounter due to its habitat preferences (Hayward et al., 2017). This suggests increased transmission rates in environments with a high cichlid species richness, which could result in more similar parasite communities. Locally, parasites in Lake Tanganyika may thus not act as a divergent, but as a convergent evolutionary force. However, the few detailed parasitological studies that exist for Lake Tanganyika hint at a huge diversity of parasite species, some of which are remarkably host-specific (Vanhove et al., 2011; Gillardin et al., 2012). Since taxonomic identification of the various metazoan macroparasites was done with a low resolution, we anticipate that our study underestimates parasite community differentiation. Divergent parasite-induced selection is therefore expected to be the predominant evolutionary force accompanying habitat adaptation and trophic specialization. A previous study on LT cichlids has shown that at early stages of diversification (e.g., among allopatric population of the same species), parasite communities are divergent, but the degree of community shifts is not related to degree of host divergence (Hablützel et al., 2016). However, as mentioned above, cases of species divergence related to changes in trophic ecology may be associated with predictable changes in parasite communities (Hablützel et al., 2017). So, while parasite community shifts may not represent a singular factor underlying host speciation, it is possible that they contribute to speciation via a reinforcement process.

### Immunogenetic diversity

An important prerequisite for a role of parasites in adaptive radiation is that the divergent parasite selection pressures lead to immunogenetic adaptation among host lineages. We therefore assessed to what extent the different cichlid tribes are immunogenetically differentiated at the level of a set of MHC genes. We also assessed to what extent habitat shifts, diet shifts, and infection patterns correlate with immunogenetic divergence. The various tribes indeed showed different MHC profiles, in particular at the amino acid level. The Limnochromini, a deep-water tribe, represented the tribe with the most divergent MHC profile, while the three largest tribes (Tropheini, Ectodini, and Lamprologini) were partially overlapping. Furthermore, immunogenetic differentiation correlated with (among others) the proportion of sand and molluscs in the diet, as well as with infection levels of nematodes and acanthocephalans. Overall, feeding on sand and infection with nematodes accounted for significant variation at the MHC level. In contrast, morphology and isotope signatures did not explain immunogenetic divergence.

These results suggest that immunogenetic differentiation in the LT cichlid radiation occurred along a similar axis as the trophic and parasitological differentiation. Yet, as with any study of macroevolution, these correlational results do not allow us to conclude whether immunogenetic differentiation has truly contributed to adaptive radiation, or whether it is merely a reflection of it. Importantly, at the micro-evolutionary level, it has been observed that both near-panmictic populations of a good disperser (*S. diagramma*) and divergent allopatric color morphs of a philopatric species (*Tropheus moorii* (Boulenger, 1898)) are immunogenetically differentiated (Hablützel et al., 2016). This suggests that immunogenetic divergence might be common, and is not exclusively linked to cases of on-going species diversification. Yet, this might as well be the case for other putative drivers of cichlid adaptive radiation, and hence it is important to further investigate the patterns that emerge from this study in detail. First, the role of sand versus rock habitat in speciation has been frequently emphasized in cichlids (Danley & Kocher, 2001). Our observation that the proportion of sand in the diet correlates with MHC divergence suggests that immunogenetic properties might be an important component of the diversification among species of these two habitat types. Second, the distinct MHC profile of the Limnochromini suggests that also the deep water selects for different immunogenetic properties. No parasitological data of the Limnochromini are currently available, except for monogenean gill-parasites of the genus *Cichlidogyrus*. Interestingly, the *Cichlidogyrus* species diversity in the Limnochromini is reduced compared to littoral cichlid hosts (Kmentová et al., 2016b). This pattern appears to be replicated in three other tribes of non-littoral cichlids, the Bathybathini, the Benthochromini, and the Trematocarini (Kmentová et al., 2016a, c). Finally, while both MHC and parasites were both correlated with specific prey items, it is intriguing that MHC variation was not explained by trophic morphology. This indicates that the trophical-morphological axes might be relatively unimportant in shaping the immunogenetic properties of LT cichlids, perhaps because of the large variety of parasites and pathogens that are not transmitted through ingestion. At the same time, we cannot exclude that a higher resolution of MHC diversity, or a larger number of cichlid species, would be required to detect a relationship between trophic morphology and immunogenetics.

In this study, we provided the first large-scale description of the MHC diversity across the major tribes of LT cichlid fishes, as previous studies were limited to small sample sizes, single tribes, species, and their populations (Klein et al., 1993; Ono et al., 1993; Málaga-Trillo et al., 1998; Blais et al., 2007; Sato et al., 2012; Hablützel et al., 2013, 2014, 2016; Hofmann et al., 2017).

As studying MHC is notoriously difficult, labor-intensive, and costly, in the past years MHC class IIB diversity has been identified using varying methods with differing efficiency and resolution. Genotyping methods such as Sanger sequencing, single-strand conformation polymorphism analysis (SSCP), restriction fragment length polymorphism (RFLP), or denaturing gradient gel electrophoresis (DGGE) (Langefors et al., 2000; Binz et al., 2001) have been replaced by amplicon sequencing using next-generation sequencing methods (Galan et al., 2010). At the time we sequenced our samples, one of these next-generation sequencing platforms, the 454-pyrosequencing, greatly surmounted the other methods in time and costs. Nevertheless, this technology is known to be error-prone, especially to PCR artifacts and sequencing errors and thus can lead to an overestimation of the actual MHC diversity (Lenz & Becker, 2008; Sommer et al., 2013). One of the strategies to circumvent the PCR artifacts in our study, such as mis-incorporation of spurious nucleotides, was the usage of a polymerase, which had 5′ → 3′ exonuclease activity and thus proofreading capabilities (Kalle et al., 2014); but see (Lenz & Becker, 2008). The 454-sequencing-specific error, the miscalling of the bases in homopolymers, had been manually corrected as the sequence length of the exon, and the expected number of amino acids was known. However, the formation of chimaeras during PCR has not been investigated and subsequently not detected in this study. The sequences of this study should not be taken as confirmed alleles as they would need further in-depth validation. Contrastingly to these biases, which could have led to an overestimation of sequence diversity, many similar alleles become indistinguishable as they are not polymorphic in the short region that is sequenced. However, comparing the minimum and maximum number of variants sequenced (1–9 per individual; Table 2), we likely have underestimated the diversity. In comparison, Málaga-Trillo et al. (1998) described up to 17 polymorphic loci with range of 1–13 alleles per individual in African cichlids, and Hofmann et al. (2017) found up to 25 alleles with an average of 12 alleles per individual.

We also expect to have a limited number of represented loci, as the usage of only one primer pair may not cover the full range of cichlid MHC loci (Málaga-Trillo et al., 1998; Murray et al., 1999; Hablützel et al., 2013; Hofmann et al., 2017). Many loci are shared across even distantly related lineages of African cichlids, resulting at a high level of trans-species polymorphisms in MHC antigen-binding sequences (Klein et al., 1993; Hablützel et al., 2013). Therefore, we expect the bias of these artifacts created by the PCR and the sequencing method to be similarly distributed across the different tribes.

While our immunogenetic results should be taken as a starting point for subsequent studies, we have shown that the chosen primer pair successfully amplified amplicons from the whole phylogenetic range of LT cichlids, and thus we were able to provide a comparative framework for an immunogenetic measurement among tribes.

## Conclusion

We showed that different cichlid tribes harbored partially non-overlapping parasite communities as well as partially non-overlapping MHC diversity. In addition, we observed various correlations between trophic ecology and morphology on the one hand, and parasite infection and immunogenetics on the other hand. Together, this implies that the potential contribution of parasites and immunogenetic adaptation to the radiation of LT cichlids should not be overlooked. In addition, it could be that habitat and diet shifts might be less important than generally accepted. Future studies should therefore consider additional candidate drivers of adaptive radiation, and investigate the potential for combined selection pressures driving adaptive radiation. To further resolve these evolutionary processes, we encourage studies that increase our knowledge of the diversity of parasites as well as immune genes within cichlid adaptive radiations.

## Electronic supplementary material

Below is the link to the electronic supplementary material.

Supplementary material 1 (PDF 545 kb)

## Data Availability

Data (i.e., tables of parasites and alignments) are available from PANGEA (10.1594/PANGAEA.893877). Raw reads are available from the Sequence Read Archive (https://www.ncbi.nlm.nih.gov/sra/SRP159021).

## References

[CR1] Acinas SG, Sarma-Rupavtarm R, Klepac-Ceraj V, Polz MF (2005). PCR-induced sequence artifacts and bias: insights from comparison of two 16S rRNA clone libraries constructed from the same sample. Applied and Environmental Microbiology.

[CR2] Adamo SA, Spiteri RJ (2009). He’s healthy, but will he survive the plague? Possible constraints on mate choice for disease resistance. Animal Behaviour.

[CR3] Baldo L, Pretus JL, Riera JL, Musilová Z, Bitja Nyom AR, Salzburger W (2017). Convergence of gut microbiotas in the adaptive radiations of African cichlid fishes. The ISME Journal.

[CR4] Beltran-Bech S, Richard F-J (2014). Impact of infection on mate choice. Animal Behaviour.

[CR5] Bernatchez L, Landry C (2003). MHC studies in nonmodel vertebrates: what have we learned about natural selection in 15 years?. Journal of Evolutionary Biology.

[CR6] Berner D, Salzburger W (2015). The genomics of organismal diversification illuminated by adaptive radiations. Trends in Genetics.

[CR7] Binz T, Reusch T, Wedekind C, Milinski M (2001). SSCP analysis of Mhc class IIB genes in the threespine stickleback. Journal of Fish Biology.

[CR8] Blais J, Rico C, van Oosterhout C, Cable J, Turner GF, Bernatchez L (2007). MHC adaptive divergence between closely related and sympatric African cichlids. PLoS ONE.

[CR9] Boecklen WJ, Yarnes CT, Cook BA, James AC (2011). On the use of stable isotopes in trophic ecology. Annual Review of Ecology Evolution and Systematics.

[CR10] Buckling A, Rainey PB (2002). The role of parasites in sympatric and allopatric host diversification. Nature.

[CR11] Cooper MD, Alder MN (2006). The evolution of adaptive immune systems. Cell.

[CR12] Danley PD, Kocher TD (2001). Speciation in rapidly diverging systems: lessons from Lake Malawi. Molecular Ecology.

[CR13] Decaestecker E, De Gersem H, Michalakis Y, Raeymaekers JAM (2013). Damped long-term host-parasite Red Queen coevolutionary dynamics: a reflection of dilution effects?. Ecology Letters.

[CR14] Dray, S., P. Legendre & G. Blanchet, 2009. packfor: Forward Selection with Permutation (Canoco p. 46). R package version 0.0-7/r58.

[CR15] Dray S, Pelissier R, Couteron P, Fortin MJ, Legendre P, Peres-Neto PR, Bellier E, Bivand R, Blanchet FG, De Caceres M, Dufour AB, Heegaard E, Jombart T, Munoz F, Oksanen J, Thioulouse J, Wagner HH (2012). Community ecology in the age of multivariate multiscale spatial analysis. Ecological Monographs.

[CR16] Eizaguirre C, Lenz TL (2010). Major histocompatibility complex polymorphism: dynamics and consequences of parasite-mediated local adaptation in fishes. Journal of Fish Biology.

[CR17] Eizaguirre C, Lenz TL, Traulsen A, Milinski M (2009). Speciation accelerated and stabilized by pleiotropic major histocompatibility complex immunogenes. Ecology Letters.

[CR19] Figueroa F, Mayer WE, Sültmann H, O’hUigin C, Tichy H, Satta Y, Takezaki N, Takahata N, Klein J (2000). Mhc class II B gene evolution in East African cichlid fishes. Immunogenetics.

[CR20] Flajnik MF, Kasahara M (2010). Origin and evolution of the adaptive immune system: genetic events and selective pressures. Nature Reviews Genetics.

[CR21] Fritz RS, Moulia C, Newcombe G (1999). Resistance of hybrid plants and animals to herbivores, pathogens, and parasites. Annual Review of Ecology and Systematics.

[CR22] Galan M, Guivier E, Caraux G, Charbonnel N, Cosson J-F (2010). A 454 multiplex sequencing method for rapid and reliable genotyping of highly polymorphic genes in large-scale studies. BMC Genomics.

[CR23] Garrigan D, Hedrick PW (2003). Perspective: detecting adaptive molecular polymorphism: lessons from the MHC. Evolution.

[CR24] Gavrilets S (2004). Fitness Landscapes and the Origin of Species.

[CR25] Gillardin C, Vanhove MM, Pariselle A, Huyse T, Volckaert FAM (2012). Ancyrocephalidae (Monogenea) of Lake Tanganyika: II: description of the first *Cichlidogyrus* spp. parasites from Tropheini fish hosts (Teleostei, Cichlidae). Parasitology Research.

[CR26] Grégoir AF, Hablützel PI, Vanhove MPM, Pariselle A, Bamps J, Volckaert FAM, Raeymaekers JAM (2015). A link between host dispersal and parasite diversity in two sympatric cichlids of Lake Tanganyika. Freshwater Biology.

[CR27] Hablützel PI, Volckaert FAM, Hellemans B, Raeymaekers JAM (2013). Differential modes of MHC class IIB gene evolution in cichlid fishes. Immunogenetics.

[CR28] Hablützel PI, Vanhove MPM, Grégoir AF, Hellemans B, Volckaert FAM, Raeymaekers JAM (2014). Intermediate number of major histocompatibility complex class IIB length variants relates to enlarged perivisceral fat deposits in the blunt-head cichlid *Tropheus moorii*. Journal of Evolutionary Biology.

[CR29] Hablützel PI, Grégoir AF, Vanhove MPM, Volckaert FAM, Raeymaekers JAM (2016). Weak link between dispersal and parasite community differentiation or immunogenetic divergence in two sympatric cichlid fishes. Molecular Ecology.

[CR30] Hablützel PI, Vanhove MPM, Deschepper P, Grégoir AF, Roose AK, Volckaert FAM, Raeymaekers JAM (2017). Parasite escape through trophic specialization in a species flock. Journal of Evolutionary Biology.

[CR31] Hamilton W, Zuk M (1982). Heritable true fitness and bright birds: a role for parasites?. Science (New York, NY).

[CR32] Hayward A, Tsuboi M, Owusu C, Kotrschal A, Buechel SD, Zidar J, Cornwallis CK, Løvlie H, Kolm N (2017). Evolutionary associations between host traits and parasite load: insights from Lake Tanganyika cichlids. Journal of Evolutionary Biology.

[CR33] Hjältén J (1998). An experimental test of hybrid resistance to insects and pathogens using *Salix caprea, S. repens* and their F1 hybrids. Oecologia.

[CR34] Hofmann MJ, Bracamonte SE, Eizaguirre C, Barluenga M (2017). Molecular characterization of MHC class IIB genes of sympatric neotropical cichlids. BMC Genetics.

[CR35] Hughes AL, Nei M (1988). Pattern of nucleotide substitution at major histocompatibility complex class I loci reveals overdominant selection. Nature.

[CR36] Hughes AL, Nei M (1989). Nucleotide substitution at major histocompatibility complex class II loci: evidence for overdominant selection. Proceedings of the National Academy of Sciences.

[CR37] Janeway C (2005). Immunobiology.

[CR38] Johnson CK, Tinker MT, Estes JA, Conrad PA, Staedler M, Miller MA, Jessup DA, Mazet JAK (2009). Prey choice and habitat use drive sea otter pathogen exposure in a resource-limited coastal system. Proceedings of the National Academy of Sciences.

[CR39] Jones DT, Taylor WR, Thornton JM (1992). The rapid generation of mutation data matrices from protein sequences. Computer Applications in the Biosciences.

[CR40] Joyce DA, Lunt DH, Genner MJ, Turner GF, Bills R, Seehausen O (2011). Repeated colonization and hybridization in Lake Malawi cichlids. Current Biology: CB.

[CR41] Kalle E, Kubista M, Rensing C (2014). Multi-template polymerase chain reaction. Biomolecular Detection and Quantification.

[CR42] Kaltz O, Shykoff JA (1998). Local adaptation in host–parasite systems. Heredity.

[CR43] Kamiya T, O’Dwyer K, Westerdahl H, Senior A, Nakagawa S (2014). A quantitative review of MHC-based mating preference: the role of diversity and dissimilarity. Molecular Ecology.

[CR44] Karvonen A, Seehausen O (2012). The role of parasitism in adaptive radiations—when might parasites promote and when might they constrain ecological speciation?. International Journal of Ecology.

[CR45] Karvonen A, Kristjánsson BK, Skúlason S, Lanki M, Rellstab C, Jokela J (2013). Water temperature, not fish morph, determines parasite infections of sympatric Icelandic threespine sticklebacks (*Gasterosteus aculeatus*). Ecology and Evolution.

[CR46] Karvonen A, Wagner CE, Selz OM, Seehausen O (2018). Divergent parasite infections in sympatric cichlid species in Lake Victoria. Journal of Evolutionary Biology.

[CR47] Katoh K, Standley DM (2013). MAFFT multiple sequence alignment software version 7: improvements in performance and usability. Molecular Biology and Evolution.

[CR48] Klein D, Ono H, O’hUigin C, Vincek V, Goldschmidt T, Klein J (1993). Extensive MHC variability in cichlid fishes of Lake Malawi. Nature.

[CR49] Klein J, Sato A, Nikolaidis N (2007). MHC, TSP, and the origin of species: from immunogenetics to evolutionary genetics. Annual Review of Genetics.

[CR50] Kmentová N, Gelnar M, Mendlová M, Van Steenberge M, Koblmüller S, Vanhove MPM (2016). Reduced host-specificity in a parasite infecting non-littoral Lake Tanganyika cichlids evidenced by intraspecific morphological and genetic diversity. Scientific Reports.

[CR51] Kmentová N, Gelnar M, Koblmüller S, Vanhove MPM (2016). First insights into the diversity of gill monogeneans of “*Gnathochromis*” and *Limnochromis* (Teleostei, Cichlidae) in Burundi: do the parasites mirror host ecology and phylogenetic history?. PeerJ.

[CR52] Kmentová N, Gelnar M, Koblmüller S, Vanhove MPM (2016). Deep-water parasite diversity in Lake Tanganyika: description of two new monogenean species from benthopelagic cichlid fishes. Parasites & Vectors.

[CR53] Knudsen R, Amundsen P-A, Klemetsen A (2010). Arctic charr in sympatry with burbot: ecological and evolutionary consequences. Hydrobiologia.

[CR54] Koblmüller S, Sefc KM, Sturmbauer C (2008). The lake Tanganyika cichlid species assemblage: recent advances in molecular phylogenetics. Hydrobiologia.

[CR55] Kocher TD (2004). Adaptive evolution and explosive speciation: the cichlid fish model. Nature Reviews Genetics.

[CR56] Kornfield I, Smith P (2000). African cichlid fishes: model systems for evolutionary biology. Annual Review of Ecology and Systematics.

[CR57] Langefors A, Lohm J, von Schantz T, Grahn M (2000). Screening of Mhc variation in Atlantic salmon (*Salmo salar*): a comparison of restriction fragment length polymorphism (RFLP), denaturing gradient gel electrophoresis (DGGE) and sequencing. Molecular Ecology.

[CR58] Legendre P, Legendre LFJ (2012). Numerical Ecology.

[CR59] Lenz TL, Becker S (2008). Simple approach to reduce PCR artefact formation leads to reliable genotyping of MHC and other highly polymorphic loci–implications for evolutionary analysis. Gene.

[CR60] Losos JB (2010). Adaptive radiation, ecological opportunity, and evolutionary determinism. The American Naturalist.

[CR61] Maan ME, Seehausen O (2011). Ecology, sexual selection and speciation. Ecology Letters.

[CR62] Maan ME, Van Rooijen A, Van Alphen J, Seehausen O (2008). Parasite-mediated sexual selection and species divergence in Lake Victoria cichlid fish. Biological Journal of the Linnean Society.

[CR63] MacColl ADC (2009). Parasites may contribute to “magic trait” evolution in the adaptive radiation of three-spined sticklebacks, *Gasterosteus aculeatus* (Gasterosteiformes: Gasterosteidae). Biological Journal of the Linnean Society.

[CR64] MacColl ADC, Chapman SM (2010). Parasites can cause selection against migrants following dispersal between environments. Functional Ecology.

[CR65] Málaga-Trillo E, Zaleska-Rutczynska Z, McAndrew B, Vincek V, Figueroa F, Sültmann H, Klein J (1998). Linkage relationships and haplotype polymorphism among cichlid Mhc class II B loci. Genetics.

[CR66] Malmstrøm M, Matschiner M, Tørresen OK, Star B, Snipen LG, Hansen TF, Baalsrud HT, Nederbragt AJ, Hanel R, Salzburger W, Stenseth NC, Jakobsen KS, Jentoft S (2016). Evolution of the immune system influences speciation rates in teleost fishes. Nature Genetics.

[CR67] Matthews B, Harmon LJ, M’Gonigle L, Marchinko KB, Schaschl H (2010). Sympatric and allopatric divergence of MHC genes in threespine stickleback. PLoS ONE.

[CR68] Meyer BS, Matschiner M, Salzburger W (2015). A tribal level phylogeny of Lake Tanganyika cichlid fishes based on a genomic multi-marker approach. Molecular Phylogenetics and Evolution.

[CR69] Meyer BS, Matschiner M, Salzburger W (2016). Disentangling incomplete lineage sorting and introgression to refine species-tree estimates for Lake Tanganyika cichlid fishes. Systematic Biology.

[CR70] Milinski M (2006). The major histocompatibility complex, sexual selection, and mate choice. Annual Review of Ecology Evolution and Systematics.

[CR71] Murray BW, Sültmann H, Klein J (1999). New family of Mhc class II A genes identified from cDNA sequences in the cichlid fish *Aulonocara hansbaenschi*. Immunogenetics.

[CR72] Muschick M, Indermaur A, Salzburger W (2012). Convergent evolution within an adaptive radiation of cichlid fishes. Current Biology: CB.

[CR103] Muschick M, Nosil P, Roesti M, Dittmann MT, Harmon L, Salzburger W (2014). Testing the stages model in the adaptive radiation of cichlid fishes in East African Lake Tanganyika. Proceedings of the Royal Society B: Biological Sciences.

[CR73] Nei M, Rooney AP (2005). Concerted and birth-and-death evolution of multigene families. Annual Review of Genetics.

[CR74] Nosil P (2012). Ecological Speciation.

[CR75] Nosil P, Vines TH, Funk DJ (2005). Perspective: reproductive isolation caused by natural selection against immigrants from divergent habitats. Evolution.

[CR76] Ono H, O’hUigin C, Tichy H, Klein J (1993). Major-histocompatibility-complex variation in two species of cichlid fishes from Lake Malawi. Molecular Biology and Evolution.

[CR77] Piertney SB, Oliver MK (2006). The evolutionary ecology of the major histocompatibility complex. Heredity.

[CR78] R Core Team (2014). R: A Language and Environment for Statistical Computing.

[CR79] Raeymaekers JAM, Hablützel PI, Grégoir AF, Bamps J, Roose AK, Vanhove MPM, Van Steenberge M, Pariselle A, Huyse T, Snoeks J, Volckaert FAM (2013). Contrasting parasite communities among allopatric colour morphs of the Lake Tanganyika cichlid *Tropheus*. BMC Evolutionary Biology.

[CR80] Rózsa L, Reiczigel J, Majoros G (2000). Quantifying parasites in samples of hosts. The Journal of Parasitology.

[CR81] Rundell R, Price T (2009). Adaptive radiation, nonadaptive radiation, ecological speciation and nonecological speciation. Trends in Ecology & Evolution.

[CR82] Salzburger W (2009). The interaction of sexually and naturally selected traits in the adaptive radiations of cichlid fishes. Molecular Ecology.

[CR102] Salzburger W (2018). Understanding explosive diversification through cichlid fish genomics. Nature Reviews Genetics.

[CR83] Salzburger W, Meyer A (2004). The species flocks of East African cichlid fishes: recent advances in molecular phylogenetics and population genetics. Die Naturwissenschaften.

[CR84] Salzburger W, Meyer A, Baric S, Verheyen E, Sturmbauer C (2002). Phylogeny of the Lake Tanganyika cichlid species flock and its relationship to the Central and East African haplochromine cichlid fish faunas. Systematic Biology.

[CR85] Salzburger W, Van Bocxlaer B, Cohen AS (2014). Ecology and evolution of the african great lakes and their faunas. Annual Review of Ecology Evolution and Systematics.

[CR86] Sato A, Dongak R, Hao L, Shintani S, Sato T (2012). Organization of Mhc class II A and B genes in the tilapiine fish Oreochromis. Immunogenetics.

[CR87] Schluter D (2000). The Ecology of Adaptive Radiation.

[CR88] Schluter D (2009). Evidence for ecological speciation and its alternative. Science (New York, NY).

[CR89] Seehausen O (2004). Hybridization and adaptive radiation. Trends in Ecology & Evolution.

[CR90] Seehausen O (2006). African cichlid fish: a model system in adaptive radiation research. Proceedings Biological sciences/The Royal Society.

[CR91] Servedio MR, Van Doorn GS, Kopp M, Frame AM, Nosil P (2011). Magic traits in speciation: “magic” but not rare?. Trends in Ecology & Evolution.

[CR92] Sommer S (2005). The importance of immune gene variability (MHC) in evolutionary ecology and conservation. Frontiers in Zoology.

[CR93] Sommer S, Courtiol A, Mazzoni CJ (2013). MHC genotyping of non-model organisms using next-generation sequencing: a new methodology to deal with artefacts and allelic dropout. BMC Genomics.

[CR94] Streelman JT, Danley P (2003). The stages of vertebrate evolutionary radiation. Trends in Ecology & Evolution.

[CR95] Summers K, McKeon S, Sellars J, Keusenkothen M, Morris J, Gloeckner D, Pressley C, Price B, Snow H (2003). Parasitic exploitation as an engine of diversity. Biological Reviews of the Cambridge Philosophical Society.

[CR96] Tamura K, Peterson D, Peterson N, Stecher G, Nei M, Kumar S (2011). MEGA5: molecular evolutionary genetics analysis using maximum likelihood, evolutionary distance, and maximum parsimony methods. Molecular Biology and Evolution.

[CR97] Vanhove MPM, Snoeks J, Volckaert FAM, Huyse T (2011). First description of monogenean parasites in Lake Tanganyika: the cichlid *Simochromis diagramma* (Teleostei, Cichlidae) harbours a high diversity of *Gyrodactylus* species (Platyhelminthes, Monogenea). Parasitology.

[CR98] Vanhove MPM, Hablützel PI, Pariselle A, Simková A, Huyse T, Raeymaekers JAM (2016). Cichlids: a host of opportunities for evolutionary parasitology. Trends in Parasitology.

[CR99] Wagner CE, Harmon LJ, Seehausen O (2012). Ecological opportunity and sexual selection together predict adaptive radiation. Nature.

[CR100] Wagner CE, Keller I, Wittwer S, Selz OM, Mwaiko S, Greuter L, Sivasundar A, Seehausen O (2013). Genome-wide RAD sequence data provide unprecedented resolution of species boundaries and relationships in the Lake Victoria cichlid adaptive radiation. Molecular Ecology.

[CR101] Wolinska J, Bittner K, Ebert D, Spaak P (2006). The coexistence of hybrid and parental *Daphnia*: the role of parasites. Proceedings Biological sciences.

