# Supplementary material for: An exploration of the links between parasites, trophic ecology, morphology, and immunogenetics in the Lake Tanganyika cichlid radiation
Source: Hydrobiologia. 2018 Oct 26;832(1):215–33. doi: 10.1007/s10750-018-3798-2 (PMC6394741; doi:10.1007/s10750-018-3798-2)
Supplement: Supplementary file 1 — Supplementary material 1 (PDF 545 kb) [file 10750_2018_3798_MOESM1_ESM.pdf]

Supplementary Material for

"An exploration of the links between parasites, trophic ecology, morphology and immunogenetics in the Lake Tanganyika cichlid radiation"

from Britta S. Meyer[1,2,3], Pascal I. Hablützel[4,5], Anna K. Roose[4], Melinda J. Hofmann[6, 7], Walter Salzburger[1] and Joost A.M. Raeymaekers[1,4,8]

[1] University of Basel, Zoological Institute, Vesalgasse 1, CH-4051 Basel, Switzerland
[2] Helmholtz Centre for Ocean Research Kiel, GEOMAR, Evolutionary Ecology of Marine Fishes, Düsternbrooker Weg 20, D-24105 Kiel, Germany
[3] present address: Max Planck Institute for Evolutionary Biology, Max Planck Research Group Behavioural Genomics, August-Thienemann-Str. 2, 24306 Plön, Germany
[4] Laboratory of Biodiversity and Evolutionary Genomics, University of Leuven, Ch. Deberiotstraat, 32, B-3000 Leuven, Belgium
[5] present address: Flanders Marine Institute, B-8400 Oostende, Belgium
[6] Dept. Biodiversity and Evolutionary Biology, Museo Nacional de Ciencias Naturales, CSIC, Calle José Gutiérrez Abascal 2, 28006, Madrid, Spain
[7] present address: Museo de Zoología, Pontificia Universidad Católica del Ecuador, Av. 12 de Octubre 1076, Quito, Ecuador
[8] present address: Faculty of Biosciences and Aquaculture, Nord University, N-8049 Bodø, Norway

Running header: Linking parasites, trophic ecology, morphology and immunogenetics
Keywords: parasites/macroparasites, trophic ecology, morphology, immunogenetics

Address for correspondence: britta-meyer@gmx.de

SUPPLEMENTARY FIGURE

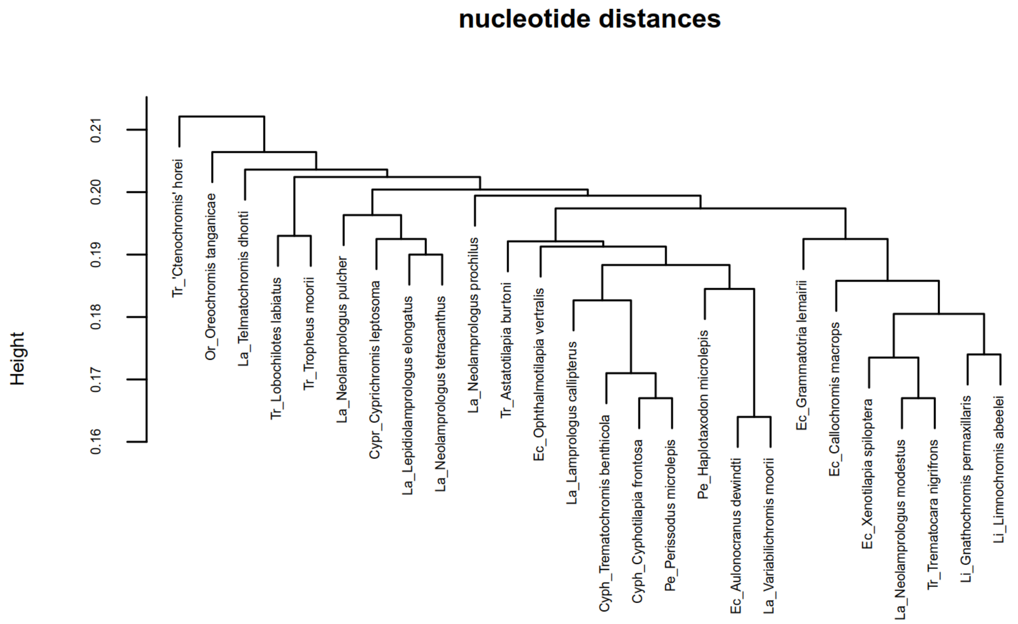

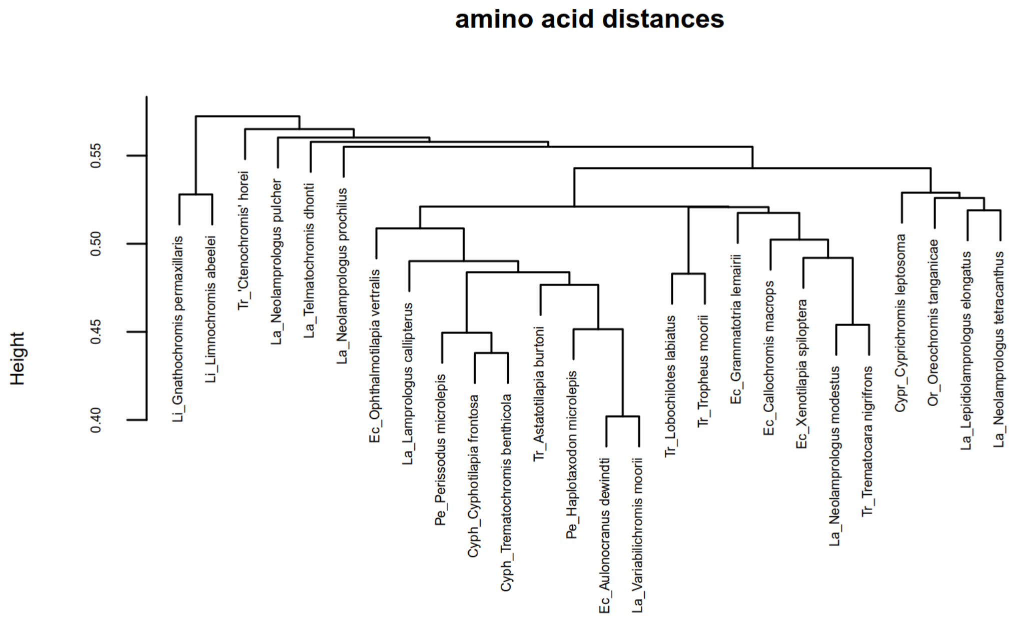

Supplementary Figure 1. UPGMA-trees for phylogenetic distances of MHC exon 2 sequences among species at the nucleotide (top) and at the amino acid level (bottom).

SUPPLEMENTARY TABLES

Supplementary Table 1. Overview of species included in the different data sets. Data by Muschick et al. (2012) include body shape, trophic morphology, diet and stable isotope data.N: sample size. 1/0: include (1) or not included (0).

Species	Tribe	Parasitology (N)	MHC (N)	Muschick (1/0)	Remarks
Trematochromis benthicola	Cyphotilapiini	0	7	1	
Cyphotilapia frontosa	Cyphotilapiini	0	16	1	
Cyprichromis leptosoma	Cyprichromini	0	11	1	
Aulonocranus dewindti	Ectodini	18	7	1	
Callochromis macrops	Ectodini	10	16	1	
Cyathopharynx furcifer	Ectodini	14	0	1	no genetic data
Grammatotria lemairii	Ectodini	0	15	1	
Ophthalmotilapia ventralis	Ectodini	14	16	1	
Xenotilapia spiloptera	Ectodini	10	16	1	
Eretmodus cyanostictus	Eretmodini	11	0	1	quality MHC data too low
Astatotilapia burtoni	Tropheini	10	8	1	parasite data incomplete; (no Gyrodactylus or fin cyst data)
Ctenochromis horei	Tropheini	10	15	1	
Gnathochromis pfefferi	Tropheini	10	0	1	quality MHC data too low
Lobochilotes labiatus	Tropheini	11	16	1	
Simochromis diagramma	Tropheini	10	0	1	no genetic data
Tropheus moorii	Tropheini	10	14	1	
Altolamprologus compressiceps	Lamprologini	11	0	1	quality MHC data too low
Julidochromis ornatus	Lamprologini	10	0	1	quality MHC data too low
Lamprologus callipterus	Lamprologini	10	14	1	
Lepidiolamprologus elongatus	Lamprologini	12	16	1	
Neolamprologus modestus	Lamprologini	10	7	1	
Neolamprologus prochilus	Lamprologini	0	5	1	
Neolamprologus pulcher	Lamprologini	10	13	1	
Neolamprologus tetracanthus	Lamprologini	11	8	1	
Telmatochromis dhonti	Lamprologini	12	14	1	no neutral genetic data
Variabilichromis moorii	Lamprologini	7	14	1	
Gnathochromis permaxillaris	Limnochromini	0	16	1	
Limnochromis abeelei	Limnochromini	0	7	1	
Oreochromis tanganicae	Oreochromini	0	7	1	
Haplotaxodon microlepis	Perrissodini	10	12	1	parasite data incomplete; (no Gyrodactylus or fin cyst data)
Perissodus microlepis	Perrissodini	10	14	1	
Trematocara nigrifrons	Trematocarini	0	12	1	

Supplementary Table 2. Spearman rank correlations across cichlid species (N = 23) between infection levels of various parasite groups and morphology, trophic ecology and isotope signatures. Prevalence (A) and mean abundance (B) vs. principal components for body shape (Body1 and Body2), lower pharyngeal jaw shape (LPJ1 and LPJ2), stable isotope signatures (δ13C and δ15N), proportional stomach and gut content (Prey1 and Prey2), and various proportional food items (m: molluscs; a: arthropods; c: crustaceans; fe: fish eggs; fs: fish scales). Correlations in bold are significant ($P < 0.05$).

(A)	Body1	Body2	LPJ 1	LPJ2	δ13c	δ15n	Prey1	Prey2	sand	aufwuchs	plants	m	a	c	fish	fe	fs
Acanthocephala	-0.3	-0.05	**0.45**	-0.09	0.39	-0.39	-0.23	-0.1	0.41	**0.44**	0.26	0.27	-0.01	-0.35	0	0.05	-0.27
Nematoda	0.1	-0.35	-0.16	0.24	-0.23	0.34	-0.04	-0.02	-0.31	0.07	-0.03	-0.4	-0.04	0.07	-0.17	-0.36	0.02
Urogyrus sp.	-0.24	-0.14	0.2	0.09	0.04	0.05	0.15	0.15	0.02	**0.53**	-0.2	0.19	0.05	0.32	0.1	0.02	0.01
Enterogyrus sp.	0.11	0.36	**-0.42**	0	-0.1	0.14	0.36	0.13	-0.29	-0.32	-0.28	0.09	0.2	0.23	**0.45**	0.3	0.02
Digenea	0.38	-0.28	0.13	0	-0.07	-0.05	-0.17	0.2	0.03	-0.22	0.06	0.23	-0.31	-0.27	-0.25	-0.01	0.13
Gill cysts	0.13	-0.13	0.11	0.01	0.15	-0.06	-0.21	-0.12	-0.09	-0.41	0.17	-0.07	0.03	0.11	**-0.46**	-0.2	-0.1
Fin cysts	0.07	**0.45**	0.07	-0.23	0.13	-0.32	-0.11	-0.11	-0.05	-0.02	0.15	0.17	0.1	-0.04	-0.35	-0.1	0.4
Gyrodactylus sp.	-0.23	0.15	-0.17	0.07	-0.32	0.08	0.32	-0.25	0	0.17	-0.11	0.1	0.31	0.03	0.38	0.02	0
Cichlidogyrus sp.	-0.09	**-0.48**	0.12	0.33	-0.11	0.11	0.15	0.29	0.36	0.14	-0.21	0.37	-0.04	0.3	-0.02	0.35	-0.22
Ergasilus sp.	-0.33	0.2	0.18	-0.03	0.19	-0.11	0.28	-0.11	-0.02	0.12	-0.13	0.32	0.39	0.15	0.08	0.03	-0.09

(B)	Body1	Body2	LPJ 1	LPJ2	δ13c	δ15n	Prey1	Prey2	sand	aufwuchs	plants	m	a	c	fish	fe	fs
Acanthocephala	-0.37	-0.25	**0.46**	0.03	0.32	-0.2	-0.22	-0.16	0.22	**0.49**	0.28	0.14	-0.01	-0.28	-0.01	0	-0.36
Nematoda	0.09	-0.36	-0.14	0.24	-0.22	0.35	-0.05	-0.02	-0.3	0.1	-0.02	-0.39	-0.04	0.08	-0.19	-0.36	-0.01
Urogyrus sp.	-0.23	-0.14	0.19	0.11	0.02	0.05	0.16	0.16	0.05	**0.52**	-0.21	0.22	0.05	0.3	0.1	0.02	0.02
Enterogyrus sp.	0.06	0.38	**-0.44**	0.05	-0.12	0.17	0.38	0.09	-0.29	-0.33	-0.28	0.1	0.22	0.19	**0.48**	0.29	-0.02
Digenea	0.37	-0.26	0.13	-0.02	-0.05	-0.05	-0.16	0.19	0.01	-0.21	0.07	0.25	-0.29	-0.26	-0.26	-0.01	0.16
Gill cysts	0.19	-0.06	0.03	0.05	0.06	-0.1	-0.15	-0.06	0.01	-0.37	0.11	0.06	0.05	0.1	**-0.46**	-0.14	0.02
Fin cysts	0.11	0.42	0.05	-0.21	0.1	-0.31	-0.09	-0.06	-0.03	-0.01	0.13	0.22	0.1	-0.03	-0.34	-0.12	0.42
Gyrodactylus sp.	-0.16	0.14	-0.17	0.01	-0.32	0.05	0.27	-0.23	0.03	0.19	-0.09	0.11	0.29	0.02	0.33	0	0.03
Cichlidogyrus sp.	-0.16	**-0.58**	0.17	0.41	-0.16	0.02	0.11	0.25	0.34	0.25	-0.15	0.27	-0.04	0.4	0.04	0.33	-0.29
Ergasilus sp.	-0.33	0.12	0.09	0.1	0.12	-0.04	**0.44**	-0.08	-0.01	0.11	-0.28	0.3	**0.48**	0.29	0.12	0.12	-0.08

Supplementary Table 3. Spearman rank correlations across cichlid species (N = 26) between dimensions of MHC divergence (MHC dimension 1 and MHC dimension 2) and morphology, trophic ecology and isotope signatures. MHC divergence at the amino acid level (A) and exon 2 level (B) vs. principal components for body shape (Body1 and Body2), lower pharyngeal jaw shape (LPJ1 and LPJ2), stable isotope signatures (δ13C and δ15N), proportional stomach and gut content (Prey1 and Prey2), and various proportional food items (m: molluscs; a: arthropods; c: crustaceans; fe: fish eggs; fs: fish scales). Correlations in bold are significant ($P < 0.05$).

(A)	Body 1	Body2	LPJ 1	LPJ2	δ13c	δ15n	Prey1	Prey2	sand	aufwuchs	plants	m	a	c	fish	fe	fs
MHC dimension 1	-0.14	0.22	-0.18	-0.07	-0.07	0.12	0.31	-0.15	0.17	0.02	-0.21	0.27	0.32	0.05	-0.19	-0.28	-0.03
MHC dimension 2	0.14	0.04	0.09	-0.19	0.31	0.14	-0.02	-0.16	**-0.43**	-0.04	0.07	**-0.39**	0.16	0.02	-0.3	-0.18	0.11
**(B)**	**Body 1**	**Body2**	**LPJ 1**	**LPJ2**	**d13c**	**d15n**	**Prey1**	**Prey2**	**sand**	**aufwuchs**	**plants**	**m**	**a**	**c**	**fish**	**fe**	**fs**
MHC dimension 1	-0.18	0.11	-0.04	0.04	-0.12	0.02	0.29	-0.13	0.3	0.02	-0.2	**0.5**	0.19	0.06	-0.03	-0.12	-0.05
MHC dimension 2	-0.06	0.11	-0.04	-0.02	0.08	0.29	0.06	-0.03	-0.25	-0.15	-0.05	-0.16	0.16	0.06	-0.29	**-0.47**	0.07

Supplementary Table 4. Spearman rank correlations across cichlid species (N = 17) between dimensions of MHC divergence (MHC dimension 1 and MHC dimension 2) and infection levels. MHC divergence at the amino acid level vs. prevalence (A) and mean abundance (B), and MHC divergence at the exon 2 level vs. prevalence (C) and mean abundance (D). Correlations in bold are significant ($P < 0.05$).

(A)	Acanthocephala	Nematoda	*Urogyrus* sp.	*Enterogyrus* sp.	Digenea	Gill cysts	Fin cysts	*Gyrodactylus* sp.	*Cichlidogyrus* sp.	*Ergasilus* sp.
MHC dimension 1	0.31	0.09	0.06	-0.35	0.09	0.32	0.23	-0.21	0.22	0.34
MHC dimension 2	-0.18	**0.73**	0.05	-0.13	-0.27	0.44	0.22	-0.34	0.07	0.14
(B)	Acanthocephala	Nematoda	*Urogyrus* sp.	*Enterogyrus* sp.	Digenea	Gill cysts	Fin cysts	*Gyrodactylus* sp.	*Cichlidogyrus* sp.	*Ergasilus* sp.
MHC dimension 1	0.25	0.08	0.11	-0.37	0.12	0.38	0.31	-0.21	0.12	0.42
MHC dimension 2	0.08	**0.75**	-0.01	-0.15	-0.27	0.36	0.16	-0.34	0.07	0.17
(C)	Acanthocephala	Nematoda	*Urogyrus* sp.	*Enterogyrus* sp.	Digenea	Gill cysts	Fin cysts	*Gyrodactylus* sp.	*Cichlidogyrus* sp.	*Ergasilus* sp.
MHC dimension 1	**0.51**	-0.34	0.01	-0.30	0.20	0.10	0.19	0.07	0.28	0.35
MHC dimension 2	-0.29	**0.71**	-0.07	-0.29	-0.10	**0.55**	0.29	-0.50	-0.03	0.01

(D)	Acanthocephala	Nematoda	*Urogyrus* sp.	*Enterogyrus* sp.	Digenea	Gill cysts	Fin cysts	*Gyrodactylus* sp.	*Cichlidogyrus* sp.	*Ergasilus* sp.
MHC dimension 1	0.34	-0.36	0.15	-0.32	0.23	0.21	0.28	0.067	0.17	0.43
MHC dimension 2	-0.11	**0.71**	-0.10	-0.30	-0.10	**0.49**	0.23	-0.50	-0.01	0.04

Supplementary Table 5. Prevalence of parasites in 23 species of Lake Tanganyika cichlids, collected along the Zambian shore of Lake Tanganyika in 2011-2013.

Species	tribe	Acanthocephala	Nematoda	Urogyrus sp.	Enterogyrus sp.	Digenea	Gill cysts	Fin cysts	Gyrodactylus sp.	Cichlidogyrus sp.	Ergasilus sp.
Altolamprologus compressiceps	Lamprologini	0.000	0.000	0.000	0.200	0.000	0.000	0.000	0.200	0.455	1.000
Astatotilapia burtoni	Tropheini	0.500	0.600	0.000	0.000	0.000	0.000			0.800	0.700
Aulonocranus dewindti	Ectodini	0.182	0.909	0.000	0.000	0.000	0.176	0.000	0.000	0.706	0.647
Callochromis macrops	Ectodini	0.600	0.000	0.000	0.000	0.400	0.000	0.000	0.000	0.900	0.900
Ctenochromis horei	Tropheini	0.200	0.200	0.100	0.000	0.000	0.100	0.000	0.000	1.000	0.500
Cyathopharynx furcifer	Ectodini	0.200	0.200	0.000	0.000	0.800	0.000	0.000	0.000	0.929	0.714
Eretmodus cyanostictus	Eretmodini	0.600	0.000	0.000	0.000	0.000	0.182	0.100	0.000	0.091	1.000
Gnathochromis pfefferi	Tropheini	0.200	0.300	0.300	0.000	0.000	0.000	0.000	0.100	0.700	1.000
Haplotaxodon microlepis	Perissodini	0.100	0.400	0.000	0.000	0.000	0.000			0.111	0.000
Julidochromis ornatus	Lamprologini	0.900	0.100	0.000	0.000	0.100	0.000	0.000	0.125	0.200	0.600
Lamprologus callipterus	Lamprologini	0.200	0.100	0.000	0.000	0.000	0.100	0.100	0.000	1.000	0.600
Lepidiolamprologus elongatus	Lamprologini	0.000	0.000	0.000	0.300	0.300	0.000	0.000	0.000	0.500	0.500
Lobochilotes labiatus	Tropheini	0.800	0.000	0.200	0.000	0.000	0.000	0.000	0.200	1.000	1.000
Neolamprologus modestus	Lamprologini	0.300	0.000	0.000	0.000	0.000	0.000	0.000	0.000	0.900	1.000
Neolamprologus pulcher	Lamprologini	0.000	0.000	0.000	0.000	0.000	0.000	0.000	0.100	0.400	0.000
Neolamprologus tetracanthus	Lamprologini	0.111	0.000	0.000	0.111	0.000	0.000	0.000	0.000	0.636	0.455
Ophthalmotilapia ventralis	Ectodini	0.300	0.000	0.000	0.000	0.600	0.143	0.000	0.000	0.786	0.357
Perissodus microlepis	Perissodini	0.000	0.250	0.000	0.000	0.500	0.100	0.000	0.000	0.700	0.500
Simochromis diagramma	Tropheini	1.000	0.000	0.000	0.000	0.000	0.000	0.000	0.000	0.400	0.300
Telmatochromis dhonti	Lamprologini	0.100	0.000	0.000	0.300	0.100	0.083	0.100	0.000	0.250	1.000
Tropheus moorii	Tropheini	0.600	0.100	0.000	0.000	0.000	0.000	0.000	0.000	0.600	0.300
Variabilichromis moorii	Lamprologini	0.000	0.429	0.143	0.000	0.000	0.000	0.143	0.000	0.000	1.000
Xenotilapia spiloptera	Ectodini	0.600	0.000	0.100	0.000	0.500	0.100	0.100	0.000	0.700	0.500

Supplementary Table 6. Mean abundance of parasites in 23 species of Lake Tanganyika cichlids, collected along the Zambian shore of Lake Tanganyika in 2011-2013

Species	tribe	Acanthocephala	Nematoda	Urogyrus sp.	Enterogyrus sp.	Digenea	Gill cysts	Fin cysts	Gyrodactylus **sp.**	Cichlidogyrus **sp.**	Ergasilus **sp.**
Altolamprologus compressiceps	Lamprologini	0.000	0.000	0.000	0.200	0.000	0.000	0.000	0.200	0.455	1000
Astatotilapia burtoni	Tropheini	0.500	0.600	0.000	0.000	0.000	0.000			0.800	0.700
Aulonocranus dewindti	Ectodini	0.182	0.909	0.000	0.000	0.000	0.176	0.000	0.000	0.706	0.647
Callochromis macrops	Ectodini	0.600	0.000	0.000	0.000	0.400	0.000	0.000	0.000	0.900	0.900
Ctenochromis horei	Tropheini	0.200	0.200	0.100	0.000	0.000	0.100	0.000	0.000	1000	0.500
Cyathopharynx furcifer	Ectodini	0.200	0.200	0.000	0.000	0.800	0.000	0.000	0.000	0.929	0.714
Eretmodus cyanostictus	Eretmodini	0.600	0.000	0.000	0.000	0.000	0.182	0.100	0.000	0.091	1000
Gnathochromis pfefferi	Tropheini	0.200	0.300	0.300	0.000	0.000	0.000	0.000	0.100	0.700	1000
Haplotaxodon microlepis	Perissodini	0.100	0.400	0.000	0.000	0.000	0.000			0.111	0.000
Julidochromis ornatus	Lamprologini	0.900	0.100	0.000	0.000	0.100	0.000	0.000	0.125	0.200	0.600
Lamprologus callipterus	Lamprologini	0.200	0.100	0.000	0.000	0.000	0.100	0.100	0.000	1000	0.600
Lepidiolamprologus elongatus	Lamprologini	0.000	0.000	0.000	0.300	0.300	0.000	0.000	0.000	0.500	0.500
Lobochilotes labiatus	Tropheini	0.800	0.000	0.200	0.000	0.000	0.000	0.000	0.200	1000	1000
Neolamprologus modestus	Lamprologini	0.300	0.000	0.000	0.000	0.000	0.000	0.000	0.000	0.900	1000
Neolamprologus pulcher	Lamprologini	0.000	0.000	0.000	0.000	0.000	0.000	0.000	0.100	0.400	0.000
Neolamprologus tetracanthus	Lamprologini	0.111	0.000	0.000	0.111	0.000	0.000	0.000	0.000	0.636	0.455
Ophthalmotilapia ventralis	Ectodini	0.300	0.000	0.000	0.000	0.600	0.143	0.000	0.000	0.786	0.357
Perissodus microlepis	Perissodini	0.000	0.250	0.000	0.000	0.500	0.100	0.000	0.000	0.700	0.500
Simochromis diagramma	Tropheini	1000	0.000	0.000	0.000	0.000	0.000	0.000	0.000	0.400	0.300
Telmatochromis dhonti	Lamprologini	0.100	0.000	0.000	0.300	0.100	0.083	0.100	0.000	0.250	1000
Tropheus moorii	Tropheini	0.600	0.100	0.000	0.000	0.000	0.000	0.000	0.000	0.600	0.300
Variabilichromis moorii	Lamprologini	0.000	0.429	0.143	0.000	0.000	0.000	0.143	0.000	0.000	1000
